# Iodine as a Heat Stress Mitigator During the Flowering Phase in Maize Plants

**DOI:** 10.3390/plants15050712

**Published:** 2026-02-27

**Authors:** Debora Teixeira Prado, Anyela Pierina Vega Quispe, Everton Geraldo de Morais, Pedro Antônio Namorato Benevenute, Leônidas Canuto dos Santos, Jucelino de Sousa Lima, Mariana Rocha de Carvalho, Paulo Eduardo Ribeiro Marchiori, Luiz Roberto Guimarães Guilherme

**Affiliations:** 1 Department of Soil Science, Federal University of Lavras, University Campus, P.O. Box 3037, Lavras 37203-202, Minas Gerais, Brazil; debora.prado@estudante.ufla.br (D.T.P.); anyela.quispe1@estudante.ufla.br (A.P.V.Q.); evertonmoraislp@gmail.com (E.G.d.M.); benevenutepedro@gmail.com (P.A.N.B.); leonidas.santos2@estudante.ufla.br (L.C.d.S.); mrocha9144@gmail.com (M.R.d.C.); 2 Department of Biology, Institute of Natural Sciences, Federal University of Lavras, University Campus, P.O. Box 3037, Lavras 37203-202, Minas Gerais, Brazil; sousajucelino12@gmail.com (J.d.S.L.); paulo.marchiori@ufla.br (P.E.R.M.)

**Keywords:** iodine fertilization, abiotic stress, phenolic compounds, chlorophyll content

## Abstract

Iodine is a non-essential element for plants, yet recent studies have shown that it plays a role in mitigating abiotic stress. Heat stress (HS) and water stress (WS) impair maize growth and development, especially during the reproductive phase. This study evaluated whether iodine applications could mitigate HS and combined HS + WS during maize flowering. The experiment was conducted under greenhouse conditions, growing maize plants in pots containing 3 kg of Oxisol. Treatments included foliar or soil applications of iodine under two stress conditions (HS and HS + WS). Iodine was applied to the soil via top dressing and as a foliar application at the start of flowering. On the last day of stress, chlorophyll levels, specific enzyme activity, compatible osmotic solutes, relative water content (RWC), and Fv:Fm (photosynthetic quantum efficiency) were measured. Grain yield was determined at the end of the crop. There was no mitigation of stress with iodine application under combined stress (HS + WS). Under HS, foliar application of iodine, compared with no iodine application mitigated stress, increasing Fv:Fm by 58% (values of 0.73 for foliar iodine application versus 0.02 for no iodine application), RWC by 83% (values of 99% for foliar iodine application), and grain yield by 35%, along with higher levels of chlorophyll *a* (+28%), chlorophyll *b* (+73%), total chlorophyll (+31%), and superoxide dismutase activity (SOD). This was also associated with a reduction in sucrose, reducing sugars, total soluble sugars, and total free amino acids. This increase in chlorophyll levels suggests greater photosynthetic capacity, while the higher SOD activity indicates a strengthened antioxidant system under HS. These mechanisms together maintain carbon assimilation and reproductive development, thereby increasing grain yield. Thus, it was concluded that iodine could help reduce HS effects during maize flowering.

## 1. Introduction

Maize (*Zea mays* L.) is considered one of the most efficient crops for biomass production, a characteristic attributed to its C4 photosynthetic pathway [1]. However, all abiotic factors, including nutrients, water, light, and temperature, must be adequately supplied to ensure that physiological processes, including photosynthesis, proceed properly, thereby guaranteeing dry matter and grain production [2,3,4]. Maize is the main food crop in many countries worldwide due to its yield potential and nutritional value [2].

Maize is a cereal highly sensitive to abiotic stress, a condition associated with low leaf plasticity and prolificacy, as well as a reduced capacity for adequate compensation [4,5,6,7,8]. Due to the impacts of global climate change, the production of various crops has been negatively affected [9]. For example, global drought stress reduces maize productivity by 40% [10]. Additionally, maize is a monoecious, diclinous plant, meaning that the male and female flowers are separate. Consequently, under water stress (WS) conditions, male flower development is favored at the expense of female flowers, resulting in delayed fertilization and increased susceptibility during the reproductive phase [11]. This asynchrony results in decreased grain set, with losses of 34% and 66% reported in maize subjected to −40 and −80 kPa during flowering and grain filling, respectively [12].

In addition to WS, heat stress (HS) has become a problem as temperatures rise. Between 1880 and 2012, the global average temperature of land and ocean surfaces increased by 0.85 °C. A further increase of around 0.2 °C is projected, indicating that stress levels are expected to rise even more in the coming years [10]. Heat stress disrupts crop growth and development [5,13,14]. Heat stress reduces both production and the levels of starch and protein in maize [5,7]. Higher temperatures during the flowering stage can reduce grain yield. This loss is primarily due to failures in pollen grain germination and pollen tube growth, as well as decreased ovule viability, as described for grain crops [15]. A study conducted on 162 inbred maize lines with different growing degree days (GDD) showed a reduction in grain yield under HS, particularly in the number of grains per ear, which decreased by 11% to 100% across the lines, regardless of GDD [16].

Under abiotic stress conditions, the biochemical and physiological mechanisms, as well as plant yield, are severely affected, and this impact can vary significantly depending on the duration, intensity, and timing of the stress. It is known that in regions with higher drought frequency (semi-arid and arid areas), the impact on crops already adapted to these conditions will eventually be less severe [17]. Abiotic stresses affect various stages of maize development and reduce crop yield to varying degrees [18].

As primary responses to WS and HS, plants develop mechanisms such as alterations in membrane fluidity and physiological processes, including the accumulation of osmolytes, the synthesis of secondary metabolites, and the activation of the antioxidant system to eliminate reactive oxygen species (ROS) [18]. Cellular dehydration is a primary effect of WS and HS. To defend against dehydration, plant cells produce osmolytes, including nucleic acids, proteins, sugars, and amino acids [13,19]. The plant also activates its antioxidant system, which includes both enzymatic and non-enzymatic components, thereby maintaining ROS balance and facilitating acclimation to abiotic stress [18].

Additionally, the plant may increase the synthesis of photosynthetic pigments to help protect the photosynthetic reaction center [18,20]. Exogenous applications of osmoprotectants, signaling molecules, and beneficial elements have shown positive effects on plants subjected to various abiotic stresses, including WS, saline, heat, and chilling stress, by modulating the mechanisms previously described [5,20,21,22,23,24,25].

Although iodine is not considered an essential element for plants, several studies have shown that it can mitigate abiotic stresses [20,23,26,27]. Among the mechanisms involved in stress mitigation through iodine application are increases in chlorophyll *a*, *b*, and total chlorophyll levels, stimulation of plant antioxidant activity, and changes in carbohydrate, sugar, and other compatible osmolyte metabolism. Consequently, reductions in ROS and lipid peroxidation help maintain photosynthetic activity and reduce productivity losses [20,21,23,28]. In this context, the study hypothesized that applying iodine could mitigate HS and WS when combined. The aims of the study were: (i) to determine the best method of iodine application to mitigate WS and HS; (ii) to assess how different modes of iodine application affect maize production, physiological parameters, and metabolic activity; (iii) to define the biochemical mechanisms involved in mitigating WS and HS through iodine application; and (iv) to demonstrate how iodine can be considered a beneficial element for plants.

## 2. Results

### 2.1. Phenotypic Responses

**Figure 1:**
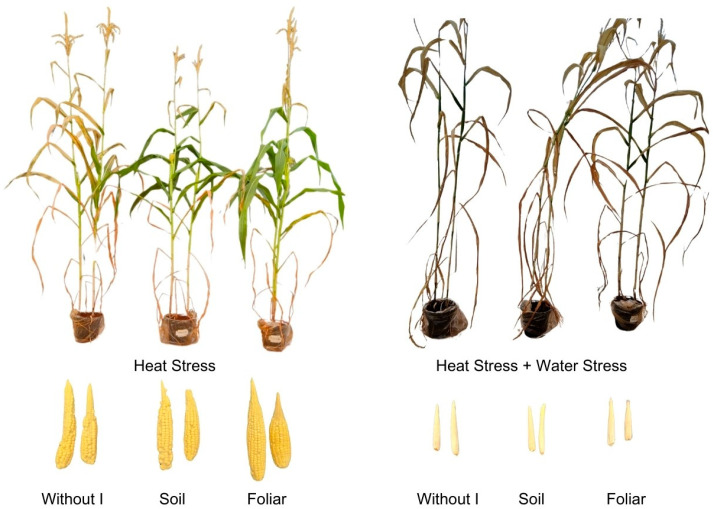
Visualization of damage stress related to the effect of iodine (I) application in different modes (without iodine application—Without I; soil iodine application—Soil; foliar iodine application—Foliar) and stress types.

Figure 1 presents the phenotypic responses of plants and visualizes maize production under HS, and HS combined with WS, across different iodine application strategies. The visual differences observed between treatments provide a comprehensive initial overview of the impact of iodine on plant performance under stress conditions. These phenotypic observations serve as a basis for subsequent physiological analyses aimed at elucidating the mechanisms underlying the observed responses. Thus, by first presenting the visible changes in the plants, we establish a logical structure that links morphological changes to the physiological parameters discussed in the following sections.

### 2.2. MINI-PAM Analysis

**Figure 2:**
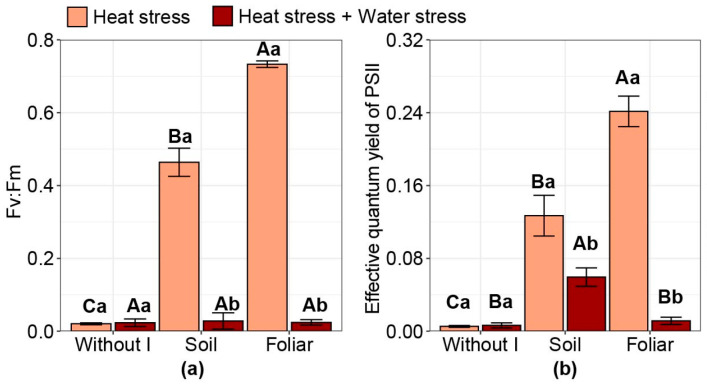
Effect of iodine application in different modes and stress types on MINI-PAM analysis: (**a**) Fv:Fm: Maximum quantum efficiency of PSII; (**b**) Effective quantum yield of photosystem II. Different uppercase letters indicate statistical differences in the modes of iodine application (without iodine application—Without I; soil iodine application—Soil; foliar iodine application—Foliar) across stress levels. In contrast, lowercase letters indicate differences in stress across iodine application modes, as determined by the Duncan test (*p* < 0.05). The bar represents the means, followed by its associated standard error.

When comparing the combination of HS and WS, plants subjected only to HS showed less severe damage, as assessed by the maximum quantum efficiency of PSII (Fv:Fm) (Figure 2a). Furthermore, iodine affected Fv:Fm values only under HS conditions. Compared with no iodine application (Fv:Fm: 0.02), Fv:Fm increased with iodine applied via soil (Fv:Fm: 0.46) and via foliar (Fv:Fm: 0.73). Foliar application increased Fv:Fm by 58% compared with soil application. The combination of HS and WS reduced the effective quantum yield of photosystem II (PSII) relative to HS alone (Figure 2b). However, under the combination of HS and WS, soil iodine application increased the effective quantum yield compared with no iodine application. Under HS alone, compared with no iodine application (values of 0.005), soil iodine application increased the effective quantum yield of PSII to 0.13, and foliar iodine application increased it to 0.24, with foliar iodine application increasing the values by 90% compared with soil iodine application.

### 2.3. Oxidative Damage and Relative Water Content

**Figure 3:**
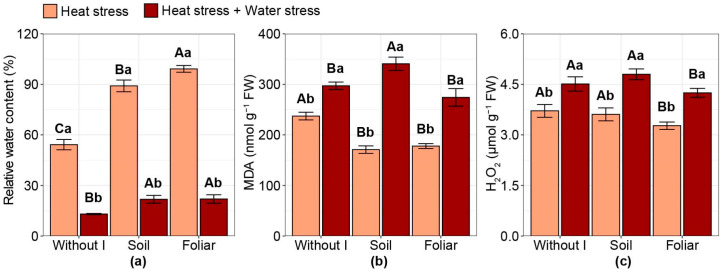
Effect of iodine application in different modes and different stress types on: (**a**) relative water content (RWC); (**b**) malondialdehyde (MDA); (**c**) hydrogen peroxide (H_2_O_2_). Different uppercase letters indicate statistical differences in the modes of iodine application (without iodine application—Without I; soil iodine application—Soil; foliar iodine application—Foliar) across stress levels. In contrast, lowercase letters indicate differences in stress across iodine application modes, as determined by the Duncan test (*p* < 0.05). The bar represents the means, followed by its associated standard error.

The relative water content (RWC) was drastically reduced, whereas malondialdehyde (MDA) content increased when HS and WS were combined compared with HS alone, regardless of the iodine application strategy (Figure 3a). Compared with no iodine application (RWC: 54%), RWC increased with soil iodine application (RWC: 89%) and foliar iodine application (RWC: 99%) for HS, with foliar iodine application increasing RWC by 83% and 11% compared with no iodine application and soil iodine application, respectively. With the combination of HS and WS, soil and foliar iodine applications increased RWC by approximately 68% compared with no iodine. However, RWC values remain low, around 22%.

There was a 19% increase in MDA content with soil iodine application compared with the average of no application and foliar iodine application under HS and WS (Figure 3b). Under HS alone, soil and foliar iodine applications reduced MDA content compared with no iodine application, resulting in a 26% decrease. For hydrogen peroxide (H_2_O_2_) content, only the foliar iodine application reduced values compared with other treatments under each stress condition, with reductions of 11% and 9% for HS alone and for the combination of HS and WS, respectively (Figure 3c).

### 2.4. Chlorophyll and Carotenoid Contents

**Figure 4:**
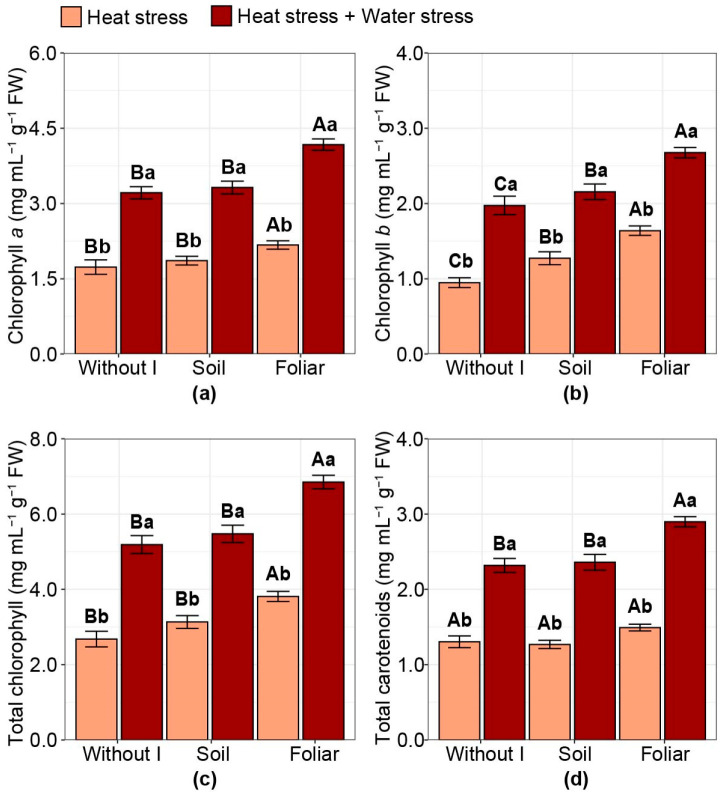
Effect of iodine application in different modes and stress types on: (**a**) chlorophyll *a* content; (**b**) chlorophyll *b *content; (**c**) total chlorophyll content; (**d**) total carotenoids content. Different uppercase letters indicate statistical differences in the modes of iodine application (without iodine application—Without I; soil iodine application—Soil; foliar iodine application—Foliar) across stress levels. In contrast, lowercase letters indicate differences in stress across iodine application modes, as determined by the Duncan test (*p* < 0.05). The bar represents the means, followed by its associated standard error.

Chlorophyll *a* content increased only with foliar iodine application compared with other treatments, depending on the stress condition evaluated, with increases of 28% and 21%, respectively, for foliar iodine application compared with an average of other treatments under HS alone and the combination of HS and WS (Figure 4a). For chlorophyll *b* content, the highest values, regardless of the stress condition evaluated, were observed with foliar iodine application, with increases of 73% and 36% under HS alone and the combined stress condition, respectively (Figure 4b). Soil iodine application also increased chlorophyll *b* content compared with no iodine application, with 34% and 9% increases under HS alone and the combined stress conditions, respectively.

Total chlorophyll content increased only with foliar iodine application compared with other treatments, and the extent of the increase depended on the stress condition evaluated (Figure 4c). Under HS alone and the combination of HS and WS, there were 31% and 28% increases in foliar iodine application compared with no iodine application and soil iodine application, respectively (Figure 4c). Carotenoid contents were influenced only by iodine under the combined stress condition, with foliar application increasing values by 24% compared with no iodine application and soil iodine application (Figure 4d). Across stress conditions, combined stress resulted in higher chlorophyll *a*, chlorophyll *b*, carotenoid, and total chlorophyll contents than HS alone, regardless of the iodine application strategy.

### 2.5. Antioxidant System Activity

**Figure 5:**
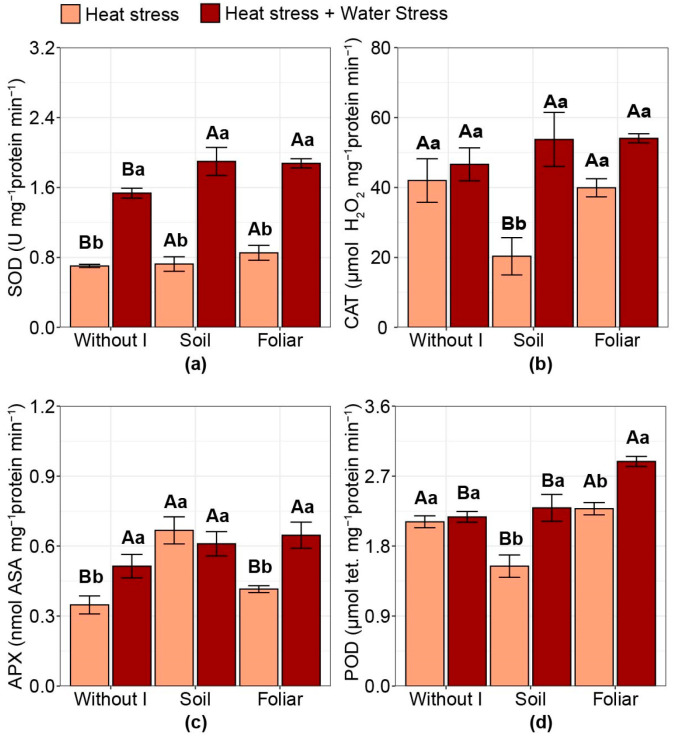
Effect of iodine application in different modes and stress types on specific antioxidant activity: (**a**) superoxide dismutase (SOD); (**b**) catalase (CAT); (**c**) ascorbate peroxidase (APX); (**d**) peroxidase (POD) (activity of tetraguaiacol). Different uppercase letters indicate statistical differences in the modes of iodine application (without iodine application—Without I; soil iodine application—Soil; foliar iodine application—Foliar) across stress levels. In contrast, lowercase letters indicate differences in stress across iodine condition applications, as determined by the Duncan test (*p* < 0.05). The bar represents the means, followed by its associated standard error.

For superoxide dismutase (SOD) activity, plants subjected to combined stress showed higher values than those subjected to HS alone (Figure 5a). Regardless of the type of stress evaluated, iodine application (both soil and foliar) increased SOD values compared with no iodine application, by 23% and 18% under HS alone and combined stress conditions, respectively. For catalase (CAT) and ascorbate peroxidase (APX) activities, iodine did not affect them under combined stress conditions (Figure 5b,c). Under HS conditions alone, soil iodine application reduced CAT values by 50% and increased APX values by 75% compared with the average of the other treatments (no iodine application and foliar iodine application) (Figure 5b,c). APX activity was higher under combined stress than under HS alone in the absence of iodine application (+48%) and lower with the foliar iodine application (−32%). Peroxidase (POD) activity decreased by 30% with soil iodine application compared with other treatments under HS alone (Figure 5d). However, under combined stress, foliar iodine application increased POD levels by 29% compared with the other treatments.

### 2.6. Starch and Compatible Osmolytes

**Figure 6:**
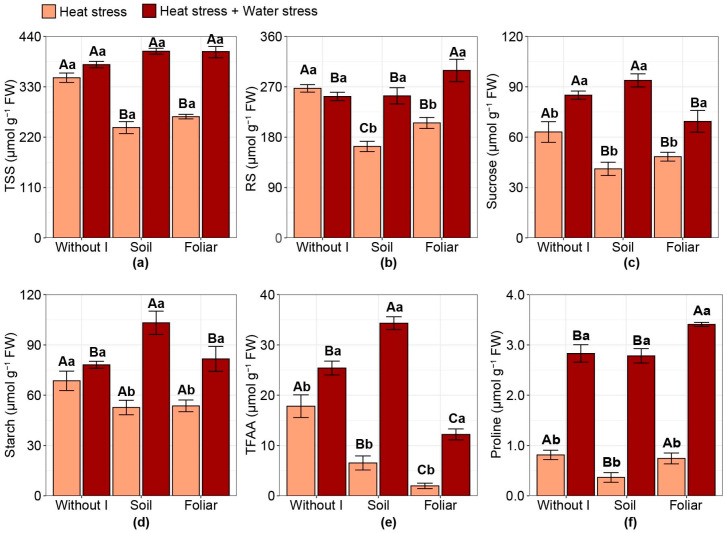
Effect of iodine application in different modes and under various stress types on: (**a**) the contents of total soluble sugar (TSS); (**b**) reducing sugars (RS); (**c**) sucrose; (**d**) starch; (**e**) total free amino acids (TFAA); (**f**) proline content. Different uppercase letters indicate statistical differences in the modes of iodine application (without iodine application—Without I; soil iodine application—Soil; foliar iodine application—Foliar) across stress levels. In contrast, lowercase letters indicate differences in stress across iodine application modes, as determined by the Duncan test (*p* < 0.05). The bar represents the means, followed by its associated standard error.

The application of iodine did not influence total soluble sugars (TSS) under the combined stress condition (Figure 6a). However, under isolated HS conditions, regardless of application mode, iodine reduced TSS by 28% compared with no iodine. Reducing sugars (RS) decreased with soil application of iodine (−39%), followed by foliar application of iodine (−23%), under HS conditions alone, compared with no application of iodine (Figure 6b). Under combined stress, only foliar iodine application increased RS values (+18%) compared with no iodine application or soil application. The combined stress condition, compared with the HS-only condition, resulted in increased sucrose content (Figure 6c). Compared with no iodine in the HS-only condition, iodine application, regardless of application method, reduced sucrose content by 29%. However, only the foliar application of iodine under combined stress reduced sucrose content by 15% compared with the other treatments.

Plants cultivated under combined stress showed higher starch content than those under HS alone when iodine was applied (Figure 6d). Under isolated HS conditions, regardless of application mode, iodine application reduced starch content by 22% compared with no iodine. Under combined stress, soil application increased starch contents by approximately 29% compared with other treatments. For total free amino acids (TFAA), combined stress increased TFAA levels compared with HS alone, regardless of the iodine application strategy (Figure 6e). Under HS alone, foliar iodine application reduced TFAA by 89%, followed by soil iodine application at −63%, compared with no iodine application. However, under combined stress, soil iodine application increased TFAA by 35%, whereas foliar iodine application reduced TFAA by 52%, compared with no iodine application. Proline content increased under combined stress compared with HS alone, regardless of the iodine application strategy (Figure 6f). Under HS alone, soil iodine application reduced proline content by 53% compared with the other treatments. However, under combined stress conditions, foliar iodine application increased proline content by 21% relative to the other treatments.

### 2.7. Biomass Production

**Figure 7:**
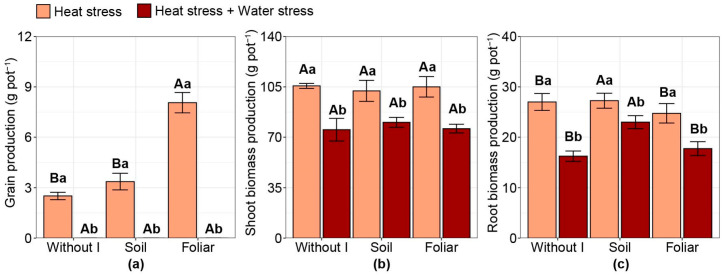
Effect of iodine application in different modes and stress types on: (**a**) grain yield; (**b**) shoot dry matter; (**c**) root dry matter. Different uppercase letters indicate statistical differences in the modes of iodine application (without iodine application—Without I; soil iodine application—Soil; foliar iodine application—Foliar) across stress levels. In contrast, lowercase letters indicate differences in stress across iodine application modes, as determined by the Duncan test (*p* < 0.05). The bar represents the means, followed by its associated standard error.

Compared with HS alone, no maize grain production was observed under combined stress (Figure 7a). Under isolated HS, only the foliar iodine application increased grain production per pot by 175% compared with the other treatments. Shoot biomass production was not influenced by the iodine strategies evaluated. However, combined stress compared with HS alone reduced shoot biomass production (Figure 7b). No interaction was observed between the factors studied and root biomass production (*p* > 0.05). It was observed that combined stress reduced root biomass production values compared with HS alone (Figure 7c). In both stress conditions, soil iodine application increased root biomass production compared with the other treatments, with increases of 5% and 35% for HS alone and combined stress conditions, respectively.

### 2.8. Multivariate Analysis

**Figure 8:**
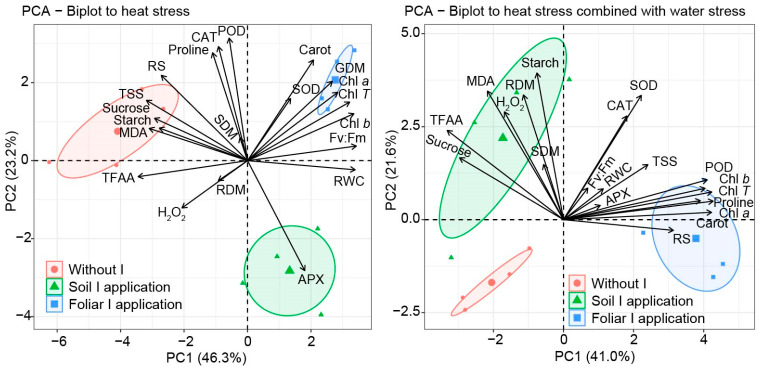
Principal component analysis of the effects of iodine application via different modes (without iodine application—Without I; soil iodine application—Soil I application; foliar iodine application—Foliar I application) under various stress conditions. Reducing sugars (RS); Total soluble solids (TSS); Sucrose; Starch; Malondialdehyde (MDA); Total free amino acids (TFAA); Hydrogen peroxide (H_2_O_2_); Relative water content (RWC); Root dry matter production (RDM); Shoot dry matter production (SDM); Grain dry matter production (GDM); Chlorophyll a (Chl *a*); Chlorophyll b (Chl *b*); Total chlorophyll (Chl *T*); Photosynthetic quantum efficiency II (Fv:Fm); Catalase (CAT); Peroxidase (POD); Ascorbate peroxidase (APX); Superoxide dismutase (SOD); Proline; Carotenoids (Carot).

In the principal component analysis (PCA) conducted on plants subjected to HS alone, a positive relation was observed between grain production and the contents of chlorophyll *a*, *b*, and total, carotenoids, SOD, Fv:Fm values, and RWC, all of which were enhanced by the foliar iodine application (Figure 8). These variables were inversely related to TSS, sucrose, starch, MDA, RS, TFAA, and H_2_O_2_, favored by the absence of iodine application. Therefore, it was found that no iodine application led to greater foliar damage (MDA and H_2_O_2_), whereas foliar iodine application reduced this damage and increased maize grain production (GDM).

In the PCA performed under HS combined with WS, a positive relationship was observed between chlorophyll *a*, *b*, and total chlorophyll; carotenoids; proline; POD; and reducing sugars, whereas a negative relationship was observed between these variables and sucrose, TFAA, MDA, H_2_O_2_, root biomass production, and starch (Figure 8). Relative water content and Fv:Fm were not related to the variables mentioned and were poorly explained by the PCA, due to the severity of the stress, which significantly reduced Fv:Fm and RWC, indicating that the combined stress prevented maize grain production.

## 3. Discussion

Regardless of the type of stress tested, photosynthetic rate decreases, but iodine can mitigate abiotic stress in maize. The adverse effects of stress may be explained by the accumulation of energy within the plant and potential cellular damage [5,8,29]. Among plant stresses, WS [6] and HS cause significant losses in maize [5,19].

Heat stress reduces maize grain yield, particularly during flowering, leading to reduced fertilization and, consequently, lower maize productivity [19,30,31]. Lobell et al. [32] found that maize yield decreased by 1% for each degree-day above 30 °C under rain conditions and 1.7% under drought conditions. In a study by Wang et al. [33], it was reported that the first short-term HS of 5 days post-ear formation resulted in the greatest yield loss, approximately −28.1%. At 15 days post-ear formation, HS reduced yield by 30.4%. In our study, this severe effect of HS and WS was confirmed by catastrophic damage to the PSII reaction center, as indicated by extremely low Fv:Fm values (0.02) (Figure 2a). Values close to zero of Fv:Fm have been reported in the literature related to high vapor pressure deficit [34,35]. The combination of HS and WS thus promotes accelerated degradation of the D1 protein, blocking electron transport and leading to extreme photoinhibition, indicating functional collapse of PSII [36,37,38].

During maize flowering, HS induces excessive respiration [39] and shortens the grain-filling phase, thereby reducing yield [5]. Water availability and temperature during anthesis and silking disrupt the synchrony between pollen release and silk emergence, a critical and sensitive phase of maize development. Water stress and HS frequently reduce pollen viability and impair silk emergence and receptivity. These negative effects prolong the interval between anthesis and silking, disrupting pollination and fertilization efficiency and, consequently, reducing grain formation and productivity under stress conditions [40], as observed in our study. However, strategies such as applying beneficial elements can mitigate these damages [3,41], as observed in our study.

Among these beneficial elements, iodine has shown high potential. We observed that iodine did not mitigate WS under HS. However, when the plants were subjected to HS alone, iodine applied via soil or foliar spray mitigated this stress, with foliar application more effective, as evidenced by increased photosystem II yield, both potential (Figure 2a) and effective (Figure 2b). This observed effect of iodine in reducing damage was observed only in HS, not in HS combined with WS, due to a critical dehydration condition, as indicated by low RWC values (~22%), suggesting yield collapse under combined stress. These results suggest that HS combined with WS exceeds the physiological window, such that under less severe stresses, iodine could attenuate stress, as shown in studies with coffee (*Coffea arabica* L.) and soybean (*Glycine max* L.) crops [20,23]. This effect can also be indicated by the higher SOD values (Figure 5a) when comparing the combined stress (HS + WS) with the isolated effect of HS. This occurs because SOD activity can play a dual role under stress, either acting protectively by dismutating the superoxide radical (O_2_•^−^) into H_2_O_2_ or, under severe stress conditions, serving as a response to elevated levels of ROS [42,43]. Its increase indicates high stress severity and a large accumulation of H_2_O_2_, as observed in our study. Thus, the low RWC and elevated SOD and H_2_O_2_ levels under combined stress indicate greater stress severity than under isolated HS.

It is extremely important to highlight that the extremely low Fv:Fm values, the drastic reduction in RWC, and the strong activation of antioxidant defenses under combined heat and humidity stress indicate that this treatment, combining HS and WS, imposed a stress intensity beyond the physiological range in which iodine can exert protective effects. Under these elevated, supraoptimal stress conditions, the beneficial effect of iodine was likely negated by the severity of the damage, which helps explain the lack of mitigation observed under combined stress in our study.

Iodine also increased leaf water content under both stress conditions, reducing oxidative stress under HS (Figure 3a). This element mitigated the WS in soybean, tomato (*Solanum lycopersicum L.*), and coffee plants treated with iodine and subjected to WS [20,23,24]. Additionally, studies using iodine for biofortification in peppers (*Capsicum annuum *L.) reduced MDA levels compared with the control, suggesting that iodine can protect cell membranes from peroxidation, as MDA is an indicator of lipid peroxidation [44].

This condition led to an increase in grain production when iodine was applied via foliar application (Figure 7a), as shown in Figure 1 and related to chlorophyll and carotenoid contents (Figure 8). These higher values for HS + WS compared with HS alone were due to a concentration effect under combined stress, as the net RWC was lower, indicating less water and thereby concentrating all cellular constituents. This concentration effect was particularly pronounced under combined stress (HS + WS), where higher chlorophyll and carotenoid levels compared to isolated HS are associated with a concentration effect resulting from reduced leaf water content and altered leaf structure, rather than a real improvement in photosynthetic capacity. The marked reduction in RWC under HS + WS indicates lower tissue hydration, which may concentrate cellular constituents by mass. Therefore, pigment accumulation under combined stress should be interpreted with caution, and future studies, including normalization by leaf area and/or leaf mass per area, would help better distinguish between concentration effects and functional physiological improvements.

Plants subjected to HS tend to reduce photosynthetic activity, leading to increased ROS production, which can cause severe damage or even death in extreme cases by inducing cellular collapse and disrupting plant metabolism [13,45]. Iodine reduced these damages among the evaluated parameters by increasing chlorophyll *a* and *b*, and carotenoid levels (Figure 4 and Figure 8). Similarly, in coffee plants, the application of 2.5 mg of KIO_3_ to the soil mitigated WS, showing a positive relationship between shoot dry mass production and chlorophyll *a*, *b*, and total chlorophyll levels [20]. Otherwise, under HS, chlorophyll content increased in response to iodine. Chlorophyll increases under HS represent a tolerance response in tomatoes [46].

This effect of iodine application in increasing chlorophyll *a* and *b* contents was observed under field conditions in rice [47] and under greenhouse conditions in coffee [20] and maize [48], an effect related to the potential of iodine to participate in photosynthesis as a component of chlorophyll and promote the synthesis of carbohydrates and proteins [49]. Plant pigments are responsible for plant coloration and are crucial for both photosynthesis and plant development [50,51]. The increase or decrease in these pigments is related to the plant’s exposure to environmental factors [7,52]. Chlorophylls are essential for photosynthesis, functioning as photoprotectants, accessory pigments, and components of complex supramolecular structures. After energy capture by the photosystems, they convert light energy into chemical energy, which is stored in simple sugars and carbohydrates through subsequent processes [50,51,53].

Authors determined that applying iodine as iodate (IO_3_^−^) or iodide (I^−^) increased the levels of APX, SOD, and CAT in lettuce, tomato, and coffee plants, with these enzymes playing a fundamental role in combating ROS [20,23,27]. Additionally, the role of iodine in forming iodinated proteins involved in photosynthetic processes through association with chloroplasts was identified [49]. Therefore, the antioxidant capacity of iodine may have favored the neutralization of ROS, which can damage chlorophyll, as evidenced by the increase in specific SOD activity, which correlated with reductions in MDA and increases in Fv:Fm and RWC values (Figure 2, Figure 3 and Figure 5). Another study with *Capsicum annuum* L. found a gradual increase in chlorophyll content with increasing iodine dose, applied via foliar spray or irrigation water [54]. These findings reinforce observations from the present study, suggesting that iodine helped maintain or increase chlorophyll content (Figure 4), which can mitigate HS.

In this study, we observed higher plant biomass when iodine was applied, primarily via foliar spray (Figure 7), which agrees with previous findings. Iodine is not considered an essential element for plant development; however, it is associated with antioxidant roles and stimulant effects [41,49,54,55,56], an antioxidant role, and stimulant effects [41,49,54,55,56]. Studies involving different iodine applications have shown that iodine enhances plant biomass by increasing the availability of iodine and nutrients in the soil [21,54,57]. In research with *Cichorium intybus* L., higher respiration rates were observed in chicory plants, contributing to enhanced metabolic activity [58]. This result suggests that iodine-treated plants may have exhibited greater metabolic activity, helping them to better cope with stress. All these findings support the study’s observations, particularly the increase in maize production (Figure 7) and the related mechanisms observed when iodine was applied (Figure 1 and Figure 8).

In a study with *Nouelia insignis* Franch, the authors found that RWC, Fv:Fm, and photosynthetic activity levels significantly decreased after 1 day of recovery from either WS or HS [59]. Other studies indicate that iodine, mainly when applied via foliar spray, enhances plant growth [54]. This study also reported increased chlorophyll content (21.5% for the best treatment), antioxidant capacity, and plant yield (Figure 4, Figure 5 and Figure 7). In the present study, an increase in these parameters was observed in the treatment that received foliar application of iodine, suggesting that iodine may have mitigated the effects of HS.

As noted in Figure 6a,b,e, iodine also reduced TFAA and RS under isolated HS, with combined stress showing higher concentrations for all macromolecules. Heat stress typically reduces amino acid and sugar content, whereas drought stress increases sugar accumulation due to growth restrictions. In tomatoes, under these combined stresses, drought effects stand out from heat effects [46]. Similarly observed here for macromolecule accumulation. A higher sucrose content is correlated with osmoprotectant function and insufficient sink activity [60], as evidenced by lower grain yields under combined stress (Figure 8). Also, it is noteworthy that these increased contents in combined stress may be due to a lower growth rate, resulting in a concentration effect.

The PCA observed that after HS, iodine application altered carbohydrate metabolism in maize plants, reducing TSS, RS, sucrose, and starch levels compared with plants without iodine (Figure 8). This change was accompanied by increased chlorophyll and carotenoid levels, which directly correlated with increased grain production in maize (Figure 4, Figure 7 and Figure 8). Our study is the first to demonstrate iodine’s beneficial effect. This effect may occur because, in plants with high photosynthetic activity, carbohydrates produced during photosynthesis are rapidly used for growth and development, leading to lower storage of carbohydrates such as TSS, reducing sugars, sucrose, and starch [14,61]. This effect occurs because the plant can redirect its metabolism to channel energy production toward growth, converting these carbohydrates into plant biomass.

The effect of iodine applied via foliar spray being more effective compared to soil application is related to the fact that iodine, when applied via foliar spray at similar or even lower doses, has greater absorption and accumulation by the leaves, resulting in lower losses due to foliar application, an effect commonly reported in experiments involving biofortification with this element [62]. However, despite this evidence, further studies should be conducted on the internal ionic status of iodine in leaves and its effects on mitigating various types of stress, which is a limitation of the present study. Furthermore, the lack of a standardized, accurate method for iodine measurement hinders assessing the internal iodine state in plants.

Although iodine acts through similar mechanisms when plants are subjected to combined HS and WS, the high stress intensity in maize plants causes significant physiological damage, affecting photosynthesis and plant growth. This result indicated that iodine could not mitigate the combined stress, which prevented grain formation and production (Figure 1). However, we emphasize that due to the cultivation conditions and the impossibility of cultivating treatment without any stress (positive control), this study has a limitation in relating the observed mitigation effects to plants grown under normal, non-stressful cultivation conditions, and in some respects, the observed effects may be considered with partial stress tolerance. Therefore, further studies should be conducted comparing plants grown with iodine application under optimal growing conditions with stressed plants, whether in greenhouse cultivation or in field conditions. Thus, these results highlight an important limitation of the present study: the combined treatment of HS and WS exceeded the physiological threshold at which iodine can protect, resulting in a collapse of photosynthetic function and productivity despite iodine application. Therefore, the results of our study should be interpreted as evidence that iodine is effective only in moderate-to-severe, but not extreme, stress scenarios, and that stress intensity is a critical factor for its protective action.

## 4. Materials and Methods

### 4.1. Experimental Conditions

The experiment was conducted in the Department of Soil Science greenhouse (21°13′33.2″ S 44°58′43.3″ W) at the Federal University of Lavras (UFLA) in Lavras, MG, Brazil. During the experiment, the greenhouse temperature was set to 28 °C ± 2 °C during the day and 15 °C ± 2 °C at night. The plants were grown in pots filled with 3 kg of soil samples (<4 mm) collected from a depth of 0.0–0.2 m, classified as Latossolo Vermelho distrófico [63], corresponding to Ferralsols [64] and Oxisols in Soil Taxonomy [65] (Table 1), being the last one adopted as the official classification in the present study. The soil density was 0.9 cm^−3^, corresponding to a soil volume of 3333 cm^3^ in the pot. The pot used had a diameter of 18 cm and a height of 20 cm.

Before the experiment, liming was performed to correct the soil pH and supply calcium (Ca) and magnesium (Mg). Liming was conducted using soil incubation with increasing doses of calcium (CaCO_3_) and magnesium (MgCO_3_) carbonates. Liming was carried out for 21 days at a 3:1 CaCO_3_:MgCO_3_ (reagent grade, Synth, Diadema, Brazil) ratio, maintaining soil moisture at 70% of the maximum water-holding capacity (MWHC). After pH correction, the soil was dried and sieved (<4 mm). After soil incubation, the achieved soil pH was 5.6 ± 0.03.

After soil correction, each experimental unit (a pot filled with soil) was sown with 10 maize (Dekalb 390 pro hybrid) seeds. Seven days after planting, plants were thinned to two per pot for the remainder of the cultivation period. Throughout the cultivation, soil moisture was maintained at 70% of the maximum water-holding capacity (MWHC). The amount of water corresponding to 70% of the MWHC was 1200 mL per pot, and this amount was monitored by randomly weighing the pots. At planting, the following nutrient quantities were provided: 135, 300, 100, 40, 0.81, 1.33, 3.66, 0.15, 5.0, and 1.55 mg kg^−1^, respectively for nitrogen (N), phosphorus (P), potassium (K), sulfur (S), boron (B), copper (Cu), manganese (Mn), molybdenum (Mo), zinc (Zn) and iron (Fe), using the following sources: NH_4_H_2_PO_4_, K_2_SO_4_, H_3_BO_3_, CuSO_4_.5H_2_O, MnSO_4_.7H_2_O, Na_2_MoO_4_, ZnSO_4_.7H_2_O and FeCl_3_.6H_2_O (reagent grade, Synth, Diadema, Brazil). Additional N fertilization was applied at 20 and 40 days after planting, providing 100 mg kg^−1^ of N per application using urea (CH_4_N_2_O) (reagent grade, Synth, Diadema, Brazil). At 35 and 45 days, 100 mg kg^−1^ of K was applied using KCl (reagent grade, Synth, Diadema, Brazil). The nutrient quantities for initial and top-dressing fertilizations were adapted from the recommendations of Novais et al. [67] for pot experiments under controlled conditions. The maize plants were cultivated for 95 days until physiological maturity and harvested in the maize phase.

### 4.2. Experimental Design and Treatments

The experiment was completely randomized with 4 replications in a 3 × 2 factorial arrangement (Table 2), totaling 6 treatments and 24 experimental units. The first factor involved iodine applications, including no iodine application, 2 kg iodine ha^−1^ applied through soil application, and 200 g iodine ha^−1^ applied through foliar application. The second factor consisted of several types of stress: HS and HS combined with WS. A treatment without stress was not carried out, as the cultivation location did not provide a stress-free environment within the greenhouse. Therefore, our aim was also to simulate field conditions with stress present.

Iodine was applied to the soil by incorporating it into urea, following methods from previous studies [48]. Urea was applied either alone or with iodine at 20 and 40 days after maize planting. The synthesis of iodine-enriched urea involved mixing urea with triethanolamine and iodine using potassium iodate (KIO_3_), along with an organic dye to confirm the proper incorporation and homogenization of iodine into urea [48]. In the soil iodine application, considering the volume of soil used, the rate per pot was 3 mg of iodine, equivalent to 1.5 mg of iodine per plant.

Foliar iodine application was conducted at the beginning of maize flowering, where 25 mL of the spray solution was applied to each experimental unit (2 plants per pot). Iodine used in foliar fertilization was also applied using KIO_3_ at a concentration of 200 g ha^−1^. The recommended maize planting density of 60,000 plants per hectare was used to calculate the amount of iodine per hectare. The iodine concentration in the foliar spray solution was calculated based on this spacing and the spray volume per experimental unit. Mineral oil was added to the foliar spray solution at 0.1% *v*/*v* to enhance spreading and improve foliar application efficiency. During the application, plants were moved to an outdoor greenhouse area to prevent further contamination of the remaining plants. In the foliar iodine application, based on the volume applied, plant density, and recommendations, 3.3 mg of iodine was applied per plant.

The decision regarding the timing between applications and the onset of different stress types was based on previous studies that found that iodine mitigates other types of stress [27]. Water stress began 7 days after the start of maize flowering (when the plants were in full bloom, 65 days), while HS was performed at 73 days after planting. Plant irrigation was completely suspended for 10 days for WS, from 65 days until 75 days after planting. During the WS, 73 days after planting, the greenhouse system was adjusted for HS, and some compartments were turned off to maintain a temperature of approximately 45 °C ± 2 °C, which was sustained for 6 h (from 07:00 a.m. to 01:00 p.m.). The temperature mentioned refers to the greenhouse’s interior, which is automatically maintained by its cooling system. This system includes a digital thermostat, and the temperature control system adjusts accordingly. The relative humidity in the greenhouse during the stress was 50%. After this period, the plants were irrigated again, and the greenhouse temperature was set to 28 °C ± 2 °C during the day and 15 °C ± 2 °C at night, returning to the previously established optimal conditions.

### 4.3. MINI-PAM Analysis and Sample Leaf Collection

At 75 days after planting (end of WS) and before irrigation, chlorophyll *a* fluorescence measurements were taken on the expanded leaf below the ear using the MINI-PAM-II Photosynthesis Yield Analyzer (Heinz Walz GmbH, Effeltrich, Germany). These analyses were conducted between 8:00 a.m. and 10:00 a.m. The MINI-PAM data included the maximum quantum efficiency of PSII (Fv:Fm) and the effective quantum yield of PSII under light-adapted conditions.

After MINI-PAM analysis, the same leaf used was collected for biochemical analyses. Half of the leaf blade was used to determine the RWC, and the other half was immediately placed in liquid N and stored at −80 °C. Subsequently, the frozen samples were individually ground to a powder in liquid N and stored at −80 °C for further analysis. In these frozen samples, the following analyses were conducted: sucrose, starch, TSS, RS, proline, total free amino acids, proteins, chlorophyll *a* and *b* contents, carotenoids, H_2_O_2_, MDA, and the activity of antioxidant enzymes: SOD, APX, CAT, and POD.

#### 4.3.1. Relative Water Content (RWC)

For the RWC measurement, 10 leaf discs were collected and weighed immediately on an analytical balance to determine fresh weight. Subsequently, the leaf discs were placed in plastic cups with distilled water (30 mL) and allowed to saturate for 24 h. After this period, filter paper was used to remove excess water, and then the discs were weighed again to determine turgid weight. Following this procedure, the samples were dried in an oven at 65 °C until constant weight to determine the dry matter [68]. The RWC was calculated using Equation (1):

(1) RWC = {[Fresh weight − Dry weight]/[Turgid weight − Dry weight]} × 100

#### 4.3.2. Chlorophyll, Macromolecules, H_2_O_2_, and MDA Content

Initially, 0.05 g of fresh leaf samples was added to microtubes for ethanol extraction. Each microtube containing 350 µL of 100% ethanol was placed in a water bath at 70–75 °C for 20 min. After this period, the samples were centrifuged at 14,000× *g* for 5 min at 4 °C. The supernatant was collected, and two additional sequential extractions were performed with 80% and 50% ethanol, respectively, following the same procedure described earlier. All collected supernatants (ethanolic extract) were stored at −20 °C for future determinations. All protocols and analyses were based on methods proposed by López-Hidalgo [69].

For pigments quantification, 25 µL of the ethanolic extract was placed in microplates and mixed with 145 µL of 100% ethanol to analyze chlorophyll *a*, chlorophyll *b*, and carotenoids. Absorbance readings were subsequently taken at 647 nm, 623 nm, and 450 nm. The final values were calculated based on the absorbance values and the fresh weight (FW) of the leaf tissues used in the extraction.

Malondialdehyde content was quantified to indicate lipid peroxidation. For this, 250 µL of the ethanolic extract and 250 µL of a solution containing 20% trichloroacetic acid and 0.5% thiobarbituric acid were added to microtubes. The microtubes were placed in a water bath at 95 °C for 30 min. Afterward, the samples were cooled on ice and centrifuged at 3000× *g* for 10 min at 4 °C. Subsequently, 150 µL of the samples was pipetted in duplicate into a microplate, and absorbance readings were taken at 532 nm and 600 nm. The MDA values were calculated from these absorbance readings [70] using Equation (2). In the ethanolic extract, the H_2_O_2_ content was determined by reacting with potassium iodide and measuring absorbance at 390 nm [71].

(2) [MDA] = (A535 − A600)/(ξ.b)

where ξ (molar extinction coefficient = 1.56 × 10^−5^ cm^−1^) and b: (optical path length = 1). Lipid peroxidation was expressed in nmol (MDA) g^−1^ of fresh weight.

Also, the RS content was determined using the DNS method from the ethanolic extract [72]. Initially, 50 µL of the ethanolic extract, 100 µL of distilled water, and 100 µL of DNS solution were added to microtubes. The tubes were placed in a water bath at 100 °C for 5 min. After cooling, the volume was adjusted to 1000 µL, and duplicate absorbance readings at 620 nm were performed. The anthrone method was used to determine the TSS content [73]. For this, 10 µL of the ethanolic extract was added to a microtube along with 320 µL of distilled water and 670 µL of anthrone solution. The microtubes were placed in a water bath at 100 °C for 3 min. After cooling, duplicate absorbance readings were taken at 620 nm. For sucrose extraction, 25 µL of the ethanolic extract and 25 µL of 30% KOH solution were combined in a microtube and heated in a water bath at 40 °C for 15 min. To determine sucrose content, 50 µL of the extract from the previous step was mixed with 280 µL of distilled water and 670 µL of anthrone solution, then placed in a water bath at 100 °C for 3 min. After cooling, duplicate absorbance readings were taken at 620 nm.

Total free amino acids were determined by mixing 50 µL of sodium citrate buffer, 50 µL of ethanolic extract, and 100 µL of ninhydrin solution (1% ninhydrin in 70% ethanol) in a microtube. Then, samples were incubated in a water bath at 95 °C for 20 min, and the absorbance was measured at 550/570 nm after cooling. Proline determination was also performed using the ninhydrin method. For this, 50 µL of the ethanolic extract was mixed with 100 µL of the reaction mix (1% ninhydrin in 60% acetic acid and 20% ethanol). The samples were then incubated in a water bath at 95 °C for 20 min. After cooling, the samples were centrifuged for 1 min at 2500× *g*, and absorbance was measured at 520 nm.

Protein extraction was performed from the pellet formed after the ethanol extraction and removal of the supernatant. For this extraction, 350 µL of 100% ethanol was added to the microtube containing the pellet. The samples were centrifuged at 14,000× *g* at 4 °C for 10 min. The samples were centrifuged again for 5 min to remove any residual alcohol. Afterward, 400 µL of 0.1 M NaOH was added to the pellet, and the samples were incubated in a water bath at 75–80 °C for 60 min. After cooling, protein quantification was performed using the Bradford method [74].

Starch extraction was performed in the same microtube (supernatant + pellet) after protein quantification. First, 70 µL of 1 M acetic acid was added and mixed, followed by 100 µL of the degradation solution (amyloglucosidase in 200 mM potassium acetate buffer, pH 4.8). The samples were then incubated in a water bath at 40 °C for 120 min. Starch quantification was performed using the anthrone method [73].

#### 4.3.3. Antioxidant Activity

The extraction of enzymes from the antioxidant system: SOD, APX, CAT and POD involved macerating 0.20 g of fresh mass in liquid nitrogen, followed by adding 1.5 mL of a buffered solution (0.1 mol L^−1^ of potassium phosphate pH 7.8, 0.1 mol L^−1^ of EDTA pH 7.0, 0.5 mol L^−1^ of DTT, 0.1 mol L^−1^ PMSF, 1 mmol L^−1^ of ascorbic acid, and 0.022 g of PVPP). The supernatant was collected after centrifugation at 13,000× *g* for 10 min at 4 °C [75]. Subsequently, the supernatant was analyzed using a microplate spectrophotometer (Epoch, BioTek Instruments, Winooski, VT, USA) according to the following methods [76,77,78,79] for SOD, APX, POD, and CAT, respectively.

### 4.4. Biomass Production

At the end of the cultivation, the maize plants were harvested and separated into roots, aboveground parts, and grains. The samples were dried at 45 °C until a constant weight was achieved, and the dry mass of the aboveground parts and roots was then determined. For grain yield determination, the dried samples were compared and adjusted to a moisture content of 13% [80].

### 4.5. Statistical Analysis

All statistical analyses were performed in R, version 4.3.0, using the agricolae (version 1.3.7), corrplot (version 0.95), factoextra (version 1.0.7), FactoMineR (version 2.11), and Metrics R (version 0.1.4) packages [81,82,83,84,85]. The basic assumptions of analysis of variance (normality, homoscedasticity, additivity, and independence of residuals) were tested and met. The F-test was significant (*p* < 0.05), and treatment means for the measured variables were differentiated using the Duncan test (*p* < 0.05) based on the absence or interaction of the factors studied. Principal component analysis was performed to evaluate correlations among variables for each stress type.

## 5. Conclusions

Iodine alleviated heat stress (HS) damage, with foliar application being more effective than soil application. The primary mechanism by which iodine mitigates HS is by increasing superoxide dismutase (SOD) activity and chlorophyll *a* and *b* contents, as well as total chlorophyll in the leaves. These increases in SOD levels reduce plasma membrane damage. Iodine increased photosynthesis in maize, as evidenced by increased photosynthetic quantum efficiency (Fv:Fm) and relative water content (RWC), and by reducing total soluble solids, sugars, and sucrose, thereby increasing grain yield. However, when plants were subjected to combined HS and WS, iodine application could not mitigate this combined stress, despite increasing chlorophyll levels, RWC, and SOD activity. This combined stress prevented the formation of maize grains. However, due to the absence of a no-stress treatment (positive control), further studies are required to verify the effect of iodine on HS mitigation by contrasting it with conditions of full and unstressed plant growth.

## Figures and Tables

**Table 1:** Chemical, physicochemical, and soil particle size distribution of the soil used.

Attributes	Values	Method ^1^
pH in water	4.5	pH at a ratio of 1:2.5 (*w*/*v*)
Soil organic matter (g kg^−1^)	24.9	Walkley-Black method
Clay (g kg^−1^)	670	Boyoucos method
Silt (g kg^−1^)	130	Boyoucos method
Sand (g kg^−1^)	200	Boyoucos method
Exchangeable calcium^2+^ (cmol_c_ kg^−1^)	0.4	1 mol L^−1^ KCl solution-soil test
Exchangeable magnesium^2+^ (cmol_c_ kg^−1^)	0.2	1 mol L^−1^ KCl solution-soil test
Available phosphorus (mg kg^−1^)	0.4	Mehlich−1 soil test
Available potassium (mg kg^−1^)	24.8	Mehlich−1 soil test
Available zinc (mg kg^−1^)	0.2	Mehlich−1 soil test
Available iron (mg kg^−1^)	38.0	Mehlich−1 soil test
Available manganese (mg kg^−1^)	3.4	Mehlich−1 soil test
Available copper (mg kg^−1^)	1.2	Mehlich−1 soil test
Available boron (mg kg^−1^)	0.01	Hot-water extraction method

^1^ The analyses were performed using methodologies already described and appropriate for the type of soil studied [66].

**Table 2:** Description of treatments used in the experiment.

Treatments	Iodine Application	Stress
T1	No iodine application	Heat stress
T2	No iodine application	Heat stress + Water stress
T3	Soil iodine application (2 kg of iodine ha^−1^)	Heat stress
T4	Soil iodine application (2 kg of iodine ha^−1^)	Heat stress + Water stress
T5	Foliar iodine application (200 g of iodine ha^−1^)	Heat stress
T6	Foliar iodine application (200 g of iodine ha^−1^)	Heat stress + Water stress

## Data Availability

The original contributions presented in this study are included in the article. Further inquiries can be directed to the corresponding author.

## References

[1] Da Silva R.G., Alves R.D.C., Zingaretti S.M. (2020). Increased [CO_2_] Causes Changes in Physiological and Genetic Responses in C4 Crops: A Brief Review. Plants.

[2] Sharma N., Rayamajhi M. (2022). Different Aspects of Weed Management in Maize (*Zea mays* L.): A Brief Review. Adv. Agric..

[3] Rajasekar M., Hussainy S.A.H., Karthik A. (2020). Effect of Moisture Deficit Conditions on the Performance of Maize (*Zea mays*): A Review. Int. J. Chem. Stud..

[4] Sheoran S., Kaur Y., Kumar S., Shukla S., Rakshit S., Kumar R. (2022). Recent Advances for Drought Stress Tolerance in Maize (*Zea mays* L.): Present Status and Future Prospects. Front. Plant Sci..

[5] El-Sappah A.H., Rather S.A., Wani S.H., Elrys A.S., Bilal M., Huang Q., Dar Z.A., Elashtokhy M.M.A., Soaud N., Koul M. (2022). Heat Stress-Mediated Constraints in Maize (*Zea mays*) Production: Challenges and Solutions. Front. Plant Sci..

[6] Jain M., Kataria S., Hirve M., Prajapati R. (2019). Water Deficit Stress Effects and Responses in Maize. Plant Abiotic Stress Tolerance.

[7] Hill C.B., Li C. (2022). Genetic Improvement of Heat Stress Tolerance in Cereal Crops. Agronomy.

[8] Salika R., Riffat J. (2021). Abiotic Stress Responses in Maize: A Review. Acta Physiol. Plant..

[9] Abbass K., Qasim M.Z., Song H., Murshed M., Mahmood H., Younis I. (2022). A Review of the Global Climate Change Impacts, Adaptation, and Sustainable Mitigation Measures. Environ. Sci. Pollut. Res..

[10] Daryanto S., Wang L., Jacinthe P.-A. (2016). Global Synthesis of Drought Effects on Maize and Wheat Production. PLoS ONE.

[11] Sangoi L., Salvador R.J. (1998). Maize Susceptibility to Drought at Flowering: A New Approach to Overcome the Problem. Ciênc. Rural.

[12] Sah R.P., Chakraborty M., Prasad K., Pandit M., Tudu V.K., Chakravarty M.K., Narayan S.C., Rana M., Moharana D. (2020). Impact of Water Deficit Stress in Maize: Phenology and Yield Components. Sci. Rep..

[13] Kan Y., Mu X.-R., Gao J., Lin H.-X., Lin Y. (2023). The Molecular Basis of Heat Stress Responses in Plants. Mol. Plant.

[14] Moore C.E., Meacham-Hensold K., Lemonnier P., Slattery R.A., Benjamin C., Bernacchi C.J., Lawson T., Cavanagh A.P. (2021). The Effect of Increasing Temperature on Crop Photosynthesis: From Enzymes to Ecosystems. J. Exp. Bot..

[15] Prasad P.V.V., Bheemanahalli R., Jagadish S.V.K. (2017). Field Crops and the Fear of Heat Stress—Opportunities, Challenges and Future Directions. Field Crops Res..

[16] Dong Z., Liu Y., Dong G., Wu H. (2021). Effect of Boiling and Frying on the Selenium Content, Speciation, and in Vitro Bioaccessibility of Selenium-Biofortified Potato (*Solanum tuberosum* L.). Food Chem..

[17] da Silva L.A.P., da Silva C.R., de Souza C.M.P., Bolfe É.L., Souza J.P.S., Leite M.E. (2023). Mapping of Aridity and Its Connections with Climate Classes and Climate Desertification in Future Scenarios—Brazilian Semi-Arid Region. Soc. Nat..

[18] Rane J., Singh A.K., Kumar M., Boraiah K.M., Meena K.K., Pradhan A., Prasad P.V.V. (2021). The Adaptation and Tolerance of Major Cereals and Legumes to Important Abiotic Stresses. Int. J. Mol. Sci..

[19] Djalovic I., Kundu S., Bahuguna R.N., Pareek A., Raza A., Singla-Pareek S.L., Prasad P.V.V., Varshney R.K. (2024). Maize and Heat Stress: Physiological, Genetic, and Molecular Insights. Plant Genome.

[20] Andrade O.V.S., de Sousa Lima J., das Neves T.T., de Morais E.G., Benevenute P.A.N., dos Santos L.C., L. Nascimento V., Guilherme L.R.G., Marchiori P.E.R. (2024). The Role of Potassium Iodate in Mitigating the Damages of Water Deficit in Coffee Plants. J. Soil Sci. Plant Nutr..

[21] Blasco B., Leyva R., Romero L., Ruiz J.M. (2013). Iodine Effects on Phenolic Metabolism in Lettuce Plants under Salt Stress. J. Agric. Food Chem..

[22] Ibrahim M.F.M., Elbar O.H.A., Farag R., Hikal M., El-Kelish A., El-Yazied A.A., Alkahtani J., El-Gawad H.G.A. (2020). Melatonin Counteracts Drought Induced Oxidative Damage and Stimulates Growth, Productivity and Fruit Quality Properties of Tomato Plants. Plants.

[23] Lima J.D.S., Andrade O.V.S., dos Santos L.C., de Morais E.G., Martins G.S., Mutz Y.S., Nascimento V.L., Marchiori P.E.R., Lopes G., Guilherme L.R.G. (2023). Soybean Plants Exposed to Low Concentrations of Potassium Iodide Have Better Tolerance to Water Deficit through the Antioxidant Enzymatic System and Photosynthesis Modulation. Plants.

[24] Lima J.D.S., Andrade O.V.S., de Morais E.G., Machado G.G.L., dos Santos L.C., de Andrade E.S., Benevenute P.A.N., Martins G.S., Nascimento V.L., Marchiori P.E.R. (2023). KI Increases Tomato Fruit Quality and Water Deficit Tolerance by Improving Antioxidant Enzyme Activity and Amino Acid Accumulation: A Priming Effect or Relief during Stress?. Plants.

[25] Rasheed R., Wahid A., Farooq M., Hussain I., Basra S.M.A. (2011). Role of Proline and Glycinebetaine Pretreatments in Improving Heat Tolerance of Sprouting Sugarcane (*Saccharum* sp.) Buds. Plant Growth Regul..

[26] Jafarian S., Sodaiezadeh H., Arani A., Hakimzadeh M.A., Sohrabizadeh Z. (2020). The Effect of Iodine On Increasing Drought Tolerance Of (*Carthamus tinctorius* L.) In Seed Germination and Early Growth Stage. J. Environ. Sci. Stud..

[27] Quispe A.P.V., de Morais E.G., Benevenute P.A.N., Lima J.D.S., dos Santos L.C., Silva M.A., Chalfun-Júnior A., Marchiori P.E.R., Guilherme L.R.G. (2025). Priming Effect with Selenium and Iodine on Broccoli Seedlings: Activation of Biochemical Mechanisms to Mitigate Cold Damages. Plant Physiol. Biochem..

[28] Zhang Y., Cao H., Wang M., Zou Z., Zhou P., Wang X., Jin J. (2023). A Review of Iodine in Plants with Biofortification: Uptake, Accumulation, Transportation, Function, and Toxicity. Sci. Total Environ..

[29] Ortez O.A., Lindsey A.J., Thomison P.R., Coulter J.A., Singh M.P., Carrijo D.R., Quinn D.J., Licht M.A., Bastos L. (2023). Corn Response to Long-term Seasonal Weather Stressors: A Review. Crop Sci..

[30] Edreira J.I.R., Mayer L.I., Otegui M.E. (2014). Heat Stress in Temperate and Tropical Maize Hybrids: Kernel Growth, Water Relations and Assimilate Availability for Grain Filling. Field Crops Res..

[31] Lizaso J.I., Ruiz-Ramos M., Rodríguez L., Gabaldon-Leal C., Oliveira J.A., Lorite I.J., Sánchez D., García E., Rodríguez A. (2018). Impact of High Temperatures in Maize: Phenology and Yield Components. Field Crops Res..

[32] Lobell D.B., Bänziger M., Magorokosho C., Vivek B. (2011). Nonlinear Heat Effects on African Maize as Evidenced by Historical Yield Trials. Nat. Clim. Chang..

[33] Wang Y., Sheng D., Zhang P., Dong X., Yan Y., Hou X., Wang P., Huang S. (2021). High Temperature Sensitivity of Kernel Formation in Different Short Periods around Silking in Maize. Environ. Exp. Bot..

[34] Murata N., Takahashi S., Nishiyama Y., Allakhverdiev S.I. (2007). Photoinhibition of Photosystem II under Environmental Stress. Biochim. Biophys. Acta (BBA)-Bioenerg..

[35] Sharma D.K., Andersen S.B., Ottosen C., Rosenqvist E. (2015). Wheat Cultivars Selected for High Fv/Fm under Heat Stress Maintain High Photosynthesis, Total Chlorophyll, Stomatal Conductance, Transpiration and Dry Matter. Physiol. Plant..

[36] Björkman O., Demmig B. (1987). Photon Yield of O_2_ Evolution and Chlorophyll Fluorescence Characteristics at 77 K among Vascular Plants of Diverse Origins. Planta.

[37] Aro E.-M., Virgin I., Andersson B. (1993). Photoinhibition of Photosystem II. Inactivation, Protein Damage and Turnover. Biochim. Biophys. Acta (BBA)-Bioenerg..

[38] Allakhverdiev S.I., Kreslavski V.D., Klimov V.V., Los D.A., Carpentier R., Mohanty P. (2008). Heat Stress: An Overview of Molecular Responses in Photosynthesis. Photosynth. Res..

[39] Guo H., Li S., Kang S., Du T., Tong L., Ding R. (2019). Annual Ecosystem Respiration of Maize Was Primarily Driven by Crop Growth and Soil Water Conditions. Agric. Ecosyst. Environ..

[40] Tang H., Zhang L., Xie X., Wang Y., Wang T., Liu C. (2025). Resilience of Maize to Environmental Stress: Insights into Drought and Heat Tolerance. Int. J. Mol. Sci..

[41] Medrano-Macías J., Leija-Martínez P., González-Morales S., Juárez-Maldonado A., Benavides-Mendoza A. (2016). Use of Iodine to Biofortify and Promote Growth and Stress Tolerance in Crops. Front. Plant Sci..

[42] Gill S.S., Tuteja N. (2010). Reactive Oxygen Species and Antioxidant Machinery in Abiotic Stress Tolerance in Crop Plants. Plant Physiol. Biochem..

[43] Foyer C.H., Noctor G. (2005). Redox Homeostasis and Antioxidant Signaling: A Metabolic Interface between Stress Perception and Physiological Responses. Plant Cell.

[44] Li R., Li D.-W., Liu H.-P., Hong C.-L., Song M.-Y., Dai Z.-X., Liu J.-W., Zhou J., Weng H.-X. (2017). Enhancing Iodine Content and Fruit Quality of Pepper (*Capsicum annuum* L.) through Biofortification. Sci. Hortic..

[45] dos Santos T.B., Ribas A.F., de Souza S.G.H., Budzinski I.G.F., Domingues D.S. (2022). Physiological Responses to Drought, Salinity, and Heat Stress in Plants: A Review. Stresses.

[46] Zhou R., Yu X., Ottosen C.-O., Rosenqvist E., Zhao L., Wang Y., Yu W., Zhao T., Wu Z. (2017). Drought Stress Had a Predominant Effect over Heat Stress on Three Tomato Cultivars Subjected to Combined Stress. BMC Plant Biol..

[47] Krzepiłko A., Kościk B., Skowrońska M., Kuśmierz S., Walczak J., Prażak R. (2022). Quality of Rye Plants (*Secale cereale*) as Affected by Agronomic Biofortification with Iodine. Plants.

[48] Cezar J.V.D.C., de Morais E.G., Lima J.D.S., Benevenute P.A.N., Guilherme L.R.G. (2024). Iodine-Enriched Urea Reduces Volatilization and Improves Nitrogen Uptake in Maize Plants. Nitrogen.

[49] Kiferle C., Martinelli M., Salzano A.M., Gonzali S., Beltrami S., Salvadori P.A., Hora K., Holwerda H.T., Scaloni A., Perata P. (2021). Evidences for a Nutritional Role of Iodine in Plants. Front. Plant Sci..

[50] Pareek S., Sagar N.A., Sharma S., Kumar V., Agarwal T., González-Aguilar G.A., Yahia E.M. (2017). Chlorophylls: Chemistry and Biological Functions. Fruit and Vegetable Phytochemicals.

[51] Pérez-Gálvez A., Viera I., Roca M. (2020). Carotenoids and Chlorophylls as Antioxidants. Antioxidants.

[52] Esteban R., Barrutia O., Artetxe U., Fernández-Marín B., Hernández A., García-Plazaola J.I. (2015). Internal and External Factors Affecting Photosynthetic Pigment Composition in Plants: A Meta-analytical Approach. New Phytol..

[53] Senge M., Ryan A., Letchford K., MacGowan S., Mielke T. (2014). Chlorophylls, Symmetry, Chirality, and Photosynthesis. Symmetry.

[54] Hassan M., Belal H., Abou-Sreea A., Rady M. (2023). Exogenous Application of Selenium or Iodine Improves the Growth, Yield and Antioxidant Status of *Capsicum annuum* L. Labyrinth Fayoum J. Sci. Interdiscip. Stud..

[55] Brown P.H., Zhao F.-J., Dobermann A. (2022). What Is a Plant Nutrient? Changing Definitions to Advance Science and Innovation in Plant Nutrition. Plant Soil.

[56] Rengel Z., Cakmak I., White P.J. (2023). Marschner’s Mineral Nutrition of Plants.

[57] Smoleń S., Sady W. (2012). Influence of Iodine Form and Application Method on the Effectiveness of Iodine Biofortification, Nitrogen Metabolism as Well as the Content of Mineral Nutrients and Heavy Metals in Spinach Plants (*Spinacia oleracea* L.). Sci. Hortic..

[58] Germ M., Kacjan-Maršić N., Kroflič A., Jerše A., Stibilj V., Golob A. (2020). Significant Accumulation of Iodine and Selenium in Chicory (*Cichorium intybus* L. Var. Foliosum Hegi) Leaves after Foliar Spraying. Plants.

[59] Zheng Y., Xia Z., Ma H., Yu Z. (2019). The Combined Effects of Water Deficit and Heat Stress on Physiological Characteristics of Endangered Nouelia Insignis. Acta Physiol. Plant..

[60] Adams W.W., Muller O., Cohu C.M., Demmig-Adams B. (2014). Photosystem II Efficiency and Non-Photochemical Fluorescence Quenching in the Context of Source-Sink Balance. Non-Photochemical Quenching and Energy Dissipation in Plants, Algae and Cyanobacteria.

[61] Baslam M., Mitsui T., Hodges M., Priesack E., Herritt M.T., Aranjuelo I., Sanz-Sáez Á. (2020). Photosynthesis in a Changing Global Climate: Scaling Up and Scaling Down in Crops. Front. Plant Sci..

[62] Lawson P.G., Daum D., Czauderna R., Meuser H., Härtling J.W. (2015). Soil versus Foliar Iodine Fertilization as a Biofortification Strategy for Field-Grown Vegetables. Front. Plant Sci..

[63] dos Santos H.G., Jacomine P.K.T., Anjos L.H.C.D., de Oliveira V.A., Lumbreras J.F., Coelho M.R., de Almeida J.A., de Araujo Filho J.C., de Oliveira J.B., Cunha T.J.F. (2018). Sistema Brasileiro de Classificação de Solos.

[64] FAO (2020). Citrus Fruit Statistical Compendium.

[65] Soil Survey Staff (2022). Keys to Soil Taxonomy.

[66] Teixeira P.C., Donagemma G.K., Fontana A., Teixeira W.G. (2017). Manual de Métodos de Análise de Solo.

[67] Novais R.F., Neves J.C.L., Barros N.F., Oliveira A.J. (1991). Ensaio Em Ambiente Controlado. Métodos de Pesquisa em Fertilidade do Solo.

[68] Barrs H.D., Weatherley P.E. (1962). A Re-Examination of the Relative Turgidity Technique for Estimating Water Deficits in Leaves. Aust. J. Biol. Sci..

[69] López-Hidalgo C., Meijón M., Lamelas L., Valledor L. (2021). The Rainbow Protocol: A Sequential Method for Quantifying Pigments, Sugars, Free Amino Acids, Phenolics, Flavonoids and MDA from a Small Amount of Sample. Plant Cell Environ..

[70] Heath R.L., Packer L. (1968). Photoperoxidation in Isolated Chloroplasts. Arch. Biochem. Biophys..

[71] Alexieva V., Sergiev I., Mapelli S., Karanov E. (2001). The Effect of Drought and Ultraviolet Radiation on Growth and Stress Markers in Pea and Wheat. Plant Cell Environ..

[72] Miller G.L. (1959). Use of Dinitrosalicylic Acid Reagent for Determination of Reducing Sugar. Anal. Chem..

[73] Chow P.S., Landhausser S.M. (2004). A Method for Routine Measurements of Total Sugar and Starch Content in Woody Plant Tissues. Tree Physiol..

[74] Bradford M.M. (1976). A Rapid and Sensitive Method for the Quantitation of Microgram Quantities of Protein Utilizing the Principle of Protein-Dye Binding. Anal. Biochem..

[75] Biemelt S., Keetman U., Albrecht G. (1998). Re-Aeration Following Hypoxia or Anoxia Leads to Activation of the Antioxidative Defense System in Roots of Wheat Seedlings. Plant Physiol..

[76] Nakano Y., Asada K. (1981). Hydrogen Peroxide Is Scavenged by Ascorbate-Specific Peroxidase in Spinach Chloroplasts. Plant Cell Physiol..

[77] Fang W.-C., Kao C.H. (2000). Enhanced Peroxidase Activity in Rice Leaves in Response to Excess Iron, Copper and Zinc. Plant Sci..

[78] Havir E.A., McHale N.A. (1987). Biochemical and Developmental Characterization of Multiple Forms of Catalase in Tobacco Leaves. Plant Physiol..

[79] Giannopolitis C.N., Ries S.K. (1977). Superoxide Dismutases: I. Occurrence in Higher Plants. Plant Physiol..

[80] Brasil (2009). Regras Para Análise de Sementes.

[81] Lê S., Josse J., Husson F. (2008). FactoMineR: An R Package for Multivariate Analysis. J. Stat. Softw..

[82] Mendiburu F. (2020). Agricolae: Statistical Procedures for Agricultural Research.

[83] R Core Team (2022). R: A Language and Environment for Statistical Computing.

[84] Wei T., Simko V. (2017). R Package “Corrplot”: Visualization of a Correlation Matrix.

[85] Kassambara A., Mundt F. (2020). Factoextra: Extract and Visualize the Results of Multivariate Data Analyses.

